# Supplementary material for: Pyruvate Dehydrogenase Kinase 1 inhibition mediated oxidative phosphorylation enhancement in cartilage promotes osteoarthritis progression
Source: BMC Musculoskelet Disord. 2023 Jul 20;24:597. doi: 10.1186/s12891-023-06585-6 (PMC10357736; doi:10.1186/s12891-023-06585-6)
Supplement: Supplementary file 1 — Additional file 1. [file 12891_2023_6585_MOESM1_ESM.zip › MF.Gsea.1653627316242/GOMF_ALCOHOL_DEHYDROGENASE_NADPPLUS_ACTIVITY.html]

Details for gene set GOMF\_ALCOHOL\_DEHYDROGENASE\_NADPPLUS\_ACTIVITY[GSEA]

|  || Dataset | 55457\_collapsed\_to\_symbols.cls.cls #OA\_versus\_normal.cls.cls #OA\_versus\_normal\_repos |
| Phenotype | cls.cls#OA\_versus\_normal\_repos |
| Upregulated in class | OA |
| GeneSet | GOMF\_ALCOHOL\_DEHYDROGENASE\_NADPPLUS\_ACTIVITY |
| Enrichment Score (ES) | 0.5417567 |
| Normalized Enrichment Score (NES) | 1.6274039 |
| Nominal p-value | 0.027027028 |
| FDR q-value | 0.13753709 |
| FWER p-Value | 0.966 |
Table: GSEA Results Summary

  

Fig 1: Enrichment plot: GOMF\_ALCOHOL\_DEHYDROGENASE\_NADPPLUS\_ACTIVITY      
 Profile of the Running ES Score & Positions of GeneSet Members on the Rank Ordered List

  

| SYMBOL | TITLE | RANK IN GENE LIST | RANK METRIC SCORE | RUNNING ES | CORE ENRICHMENT || 1 | DHRS7 | dehydrogenase/reductase 7 [Source:HGNC Symbol;Acc:HGNC:21524] | 45 | 1.005 | 0.2811 | Yes |
| 2 | AKR1A1 | aldo-keto reductase family 1 member A1 [Source:HGNC Symbol;Acc:HGNC:380] | 808 | 0.567 | 0.3826 | Yes |
| 3 | AKR1C3 | aldo-keto reductase family 1 member C3 [Source:HGNC Symbol;Acc:HGNC:386] | 1996 | 0.361 | 0.3928 | Yes |
| 4 | AKR7A2 | aldo-keto reductase family 7 member A2 [Source:HGNC Symbol;Acc:HGNC:389] | 2690 | 0.280 | 0.4184 | Yes |
| 5 | DHRS4 | dehydrogenase/reductase 4 [Source:HGNC Symbol;Acc:HGNC:16985] | 2708 | 0.279 | 0.4960 | Yes |
| 6 | AKR1B1 | aldo-keto reductase family 1 member B [Source:HGNC Symbol;Acc:HGNC:381] | 3138 | 0.236 | 0.5297 | Yes |
| 7 | RDH14 | retinol dehydrogenase 14 [Source:HGNC Symbol;Acc:HGNC:19979] | 3952 | 0.160 | 0.5120 | Yes |
| 8 | RDH11 | retinol dehydrogenase 11 [Source:HGNC Symbol;Acc:HGNC:17964] | 4102 | 0.146 | 0.5418 | Yes |
| 9 | DHRS3 | dehydrogenase/reductase 3 [Source:HGNC Symbol;Acc:HGNC:17693] | 4770 | 0.089 | 0.5152 | No |
| 10 | AKR1D1 | aldo-keto reductase family 1 member D1 [Source:HGNC Symbol;Acc:HGNC:388] | 5836 | 0.006 | 0.4344 | No |
| 11 | AKR1C4 | aldo-keto reductase family 1 member C4 [Source:HGNC Symbol;Acc:HGNC:387] | 5843 | 0.005 | 0.4354 | No |
| 12 | RDH8 | retinol dehydrogenase 8 [Source:HGNC Symbol;Acc:HGNC:14423] | 5947 | -0.001 | 0.4278 | No |
| 13 | ALDH3A1 | aldehyde dehydrogenase 3 family member A1 [Source:HGNC Symbol;Acc:HGNC:405] | 6413 | -0.036 | 0.4019 | No |
| 14 | AKR1C1 | aldo-keto reductase family 1 member C1 [Source:HGNC Symbol;Acc:HGNC:384] | 8198 | -0.171 | 0.3119 | No |
| 15 | AKR1B10 | aldo-keto reductase family 1 member B10 [Source:HGNC Symbol;Acc:HGNC:382] | 8420 | -0.187 | 0.3477 | No |
Table: GSEA details [plain text format]

  

Fig 2: GOMF\_ALCOHOL\_DEHYDROGENASE\_NADPPLUS\_ACTIVITY      
 Blue-Pink O' Gram in the Space of the Analyzed GeneSet

  

Fig 3: GOMF\_ALCOHOL\_DEHYDROGENASE\_NADPPLUS\_ACTIVITY: Random ES distribution      
 Gene set null distribution of ES for **GOMF\_ALCOHOL\_DEHYDROGENASE\_NADPPLUS\_ACTIVITY**
